# Supplementary material for: Designing efficient genetic code expansion in Bacillus subtilis to gain biological insights
Source: Nat Commun. 2021 Sep 14;12:5429. doi: 10.1038/s41467-021-25691-4 (PMC8440579; doi:10.1038/s41467-021-25691-4)
Supplement: Supplementary file 1 — Supplementary Information [file 41467_2021_25691_MOESM1_ESM.pdf]

Supplemental Text

Promoter Screen

Five different combinations of AARS and tRNA promoter were constructed and tested for activity, as it has been previously demonstrated that optimization of expression levels is necessary for good activity[1]. These combinations were tested and a pVeg constitutive promoter in front of the synthetase and a pSer promoter in front of the tRNA were found to be the most effective (Supp. Fig. 1A).

Reporter optimization

Initial experiments with the mNeongreen reporter expressed by the IPTG-inducible pHyperspank showed high levels of background in the absence of nsAA & synthetase. Follow-up experiments indicated that the background was due to a secondary start codon at Methionine10 of mNeongreen, driven by a ribosomal binding site ~1/3 of the strength of the canonical pHyperspank contained in residues 4-8 (Supp. Fig. 1B-C). Ribosomal binding site presence and strength was calculated from the Salis lab ribosomal binding site calculator[2]. These findings could drive reinterpretation of subcellular microscopy experiments that used C-terminal or sandwich fusion mNeongreen tags[3,4], as the commonly used mNeongreen sequence is capable of initiating translation independently. An M10S mutation suppressed the background and reported 30-50 fold increase in mNeongreen fluorescence upon addition of the nsAA (Supp. Fig. 1C). All subsequent usage of mNeongreen used the M10S variant.

Synthetase Promiscuity

Further exploration of general nsAA incorporation in *B. subtilis* with the extremely sensitive nanoluciferase reporter[5] showed subtleties of incorporation efficiency and promiscuous background incorporation. The MjTyrRSs are known to promiscuously incorporate native amino acids in the absence of the target nsAAs[6], which is reflected here by some background incorporation in the absence of nsAA. When tested with the TAG-nanoluciferase reporter, the presence of napARS increased TAG-luciferase expression 22-fold in the absence of nsAA. The addition of nsAA increased expression another 25-fold, for a total of 556-fold increase over the TAG-nanoluciferase reporter alone. Both the ScwRS and MaPylRS showed minimal background incorporation, with addition of the synthetase causing 1.3 and 3.6-fold over the reporter in the absence of nsAA, respectively. Addition of nsAA increased expression 11 and 295-fold over the reporter in the presence of corresponding nsAAs, respectively (Supp. Figure 1G).

nsAA concentration

In general, nsAAs are used at 1 mM concentration. However, some AARSs are capable of functioning effectively at lower concentrations, especially the MjTyrRS-based synthetases. In this study, we found 1 mM to be necessary for all non-tyrosine-based nsAAs and nsAA 3, likely due to its structural distance from the canonical phenyl-group common to other tyrosine-based nsAAs. However, the other tyrosine nsAAs were used at 100 uM as they retained full activity at that level. Some nsAAs cannot be used at 1 mM, especially nsAA 1, which precipitates above 500 uM in LB, and BiPyrA, which causes toxicity at 1 mM. This toxicity may be due to the metal-chelating nature of this nsAA, though we have done no experiments to confirm this.

Supplemental Figures

Supplemental Figure 1

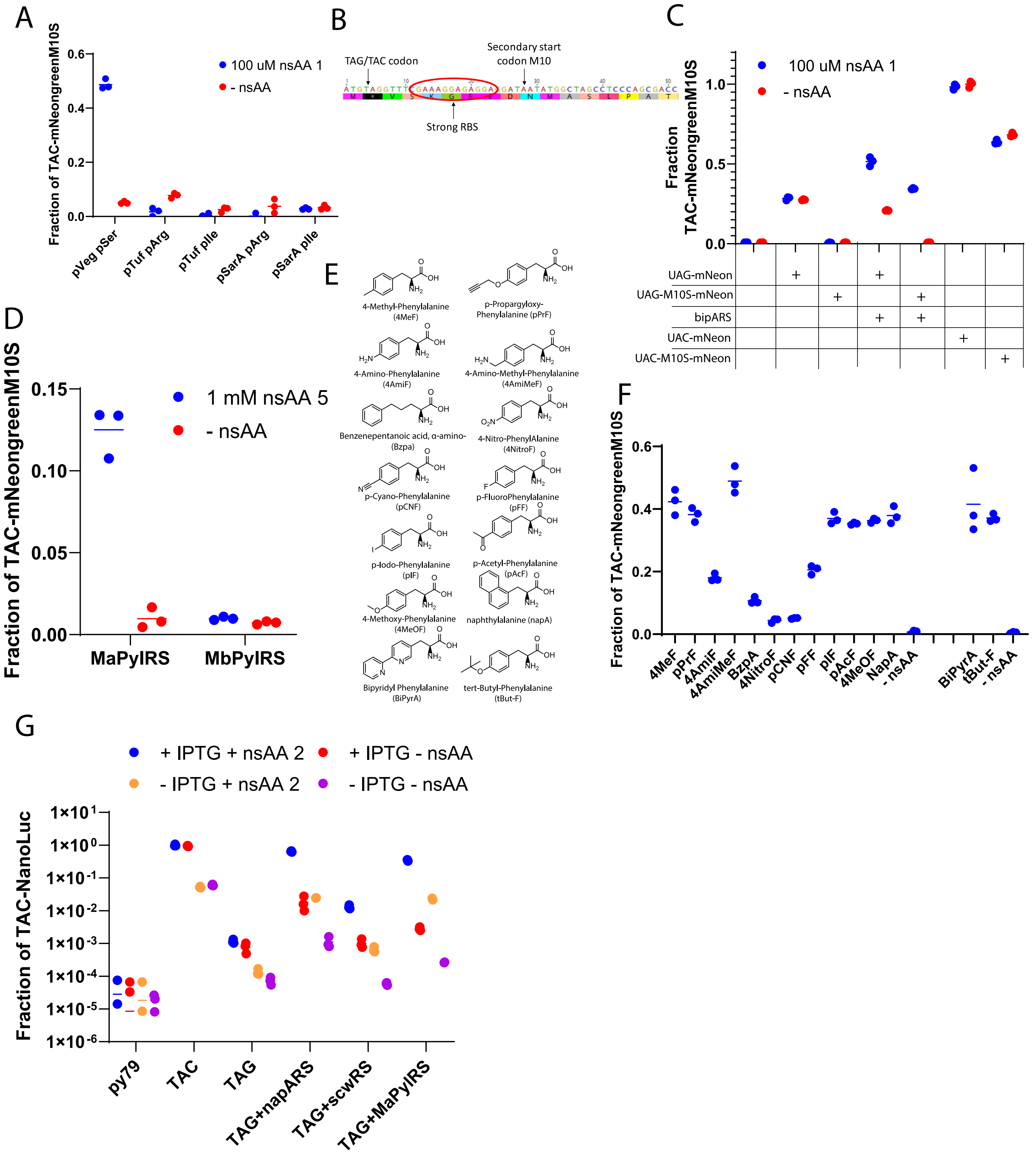

Supplemental Figure 1: ***Extended nsAA incorporation in B. subtilis*****.** For all graphs three replicates are shown with a horizontal bar at mean. 100 uM of nsAA was used for all tyrosine-based nsAAs, but 1 mM was used for nsAAs **3, 5** & **6** A). Assay of 5 AARS/tRNA promoter combinations, with the identity of the promoter indicated below the bars. Reported by an pHyperspank-inducible mNeongreen containing a UAG codon in an N-terminal linker and normalized to maximum fluorescence from the experiment. B) Schematic of first 17 residues of TAG-mNeongreen reporter, with the M10 capable of secondary translational start indicated. Secondary RBS strength was calculated as approximately 1/3 the strength of the pHyperspank RBS with the

Salis Lab Ribosomal Biding site calculator. C) Assay of the mNeongreen and the M10S mNeongreen reporters with and without associated synthetase. The reported TAG or TAC codon is at position 2, after the start methionine. D) Comparison of *B. subtilis* activity of MaPylRS and MbPylRS activity with the TAG-M10SmNeongreen reporter, normalized to TAC-M10SmNeongreen. MbPylRS is homologous . E) Structures and names of additional nsAAs incorporated in F) using the napARS synthetase (bars on left) or the bipARS synthetase (bars on right). G) Assay of 3 different synthetases incorporating nsAA **1** for napARS, **5** for MaPylRS and **6** for ScwRS using a sensitive IPTG-inducible TAG-nanoluciferase reporter capable of reporting over 5 orders of magnitude. Log plot of pHyperspank-Nanoluciferase levels with a TAG and a TAC codon to demonstrate accurate levels of promiscuity vs. native amino acids.

Supplemental Figure 2

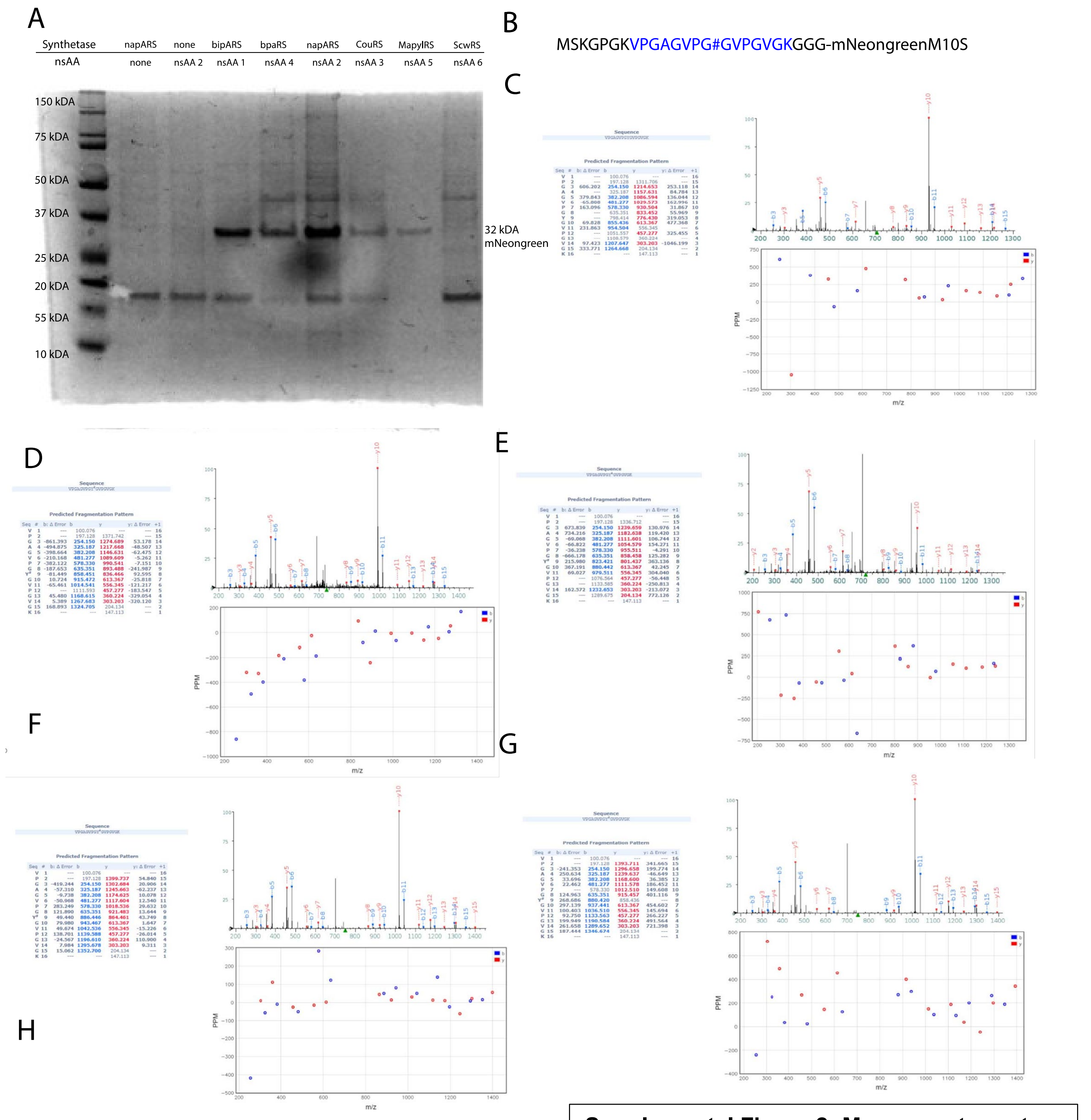

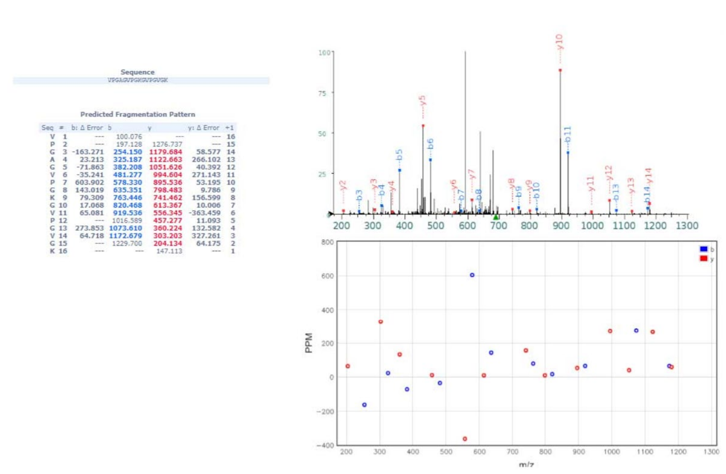

Supplemental Figure 2: Mass-spectrometry of nsAA-containing peptides. A) Gel image showing purified mNeongreen containing nsAAs at 32 kDA. B) Sequence of Elastin-like region of reporter, with the peptide shown in mass spec marked in blue and position of nsAA indicated by #.C-H) Mass Spectrums of peptides containing C) Tyrosine D) nsAA 2 E) nsAA 1 F) nsAA 4 G) nsAA 3 H) Lysine (likely resulting from deprotection of nsAA 5)

Supplemental Figure 3

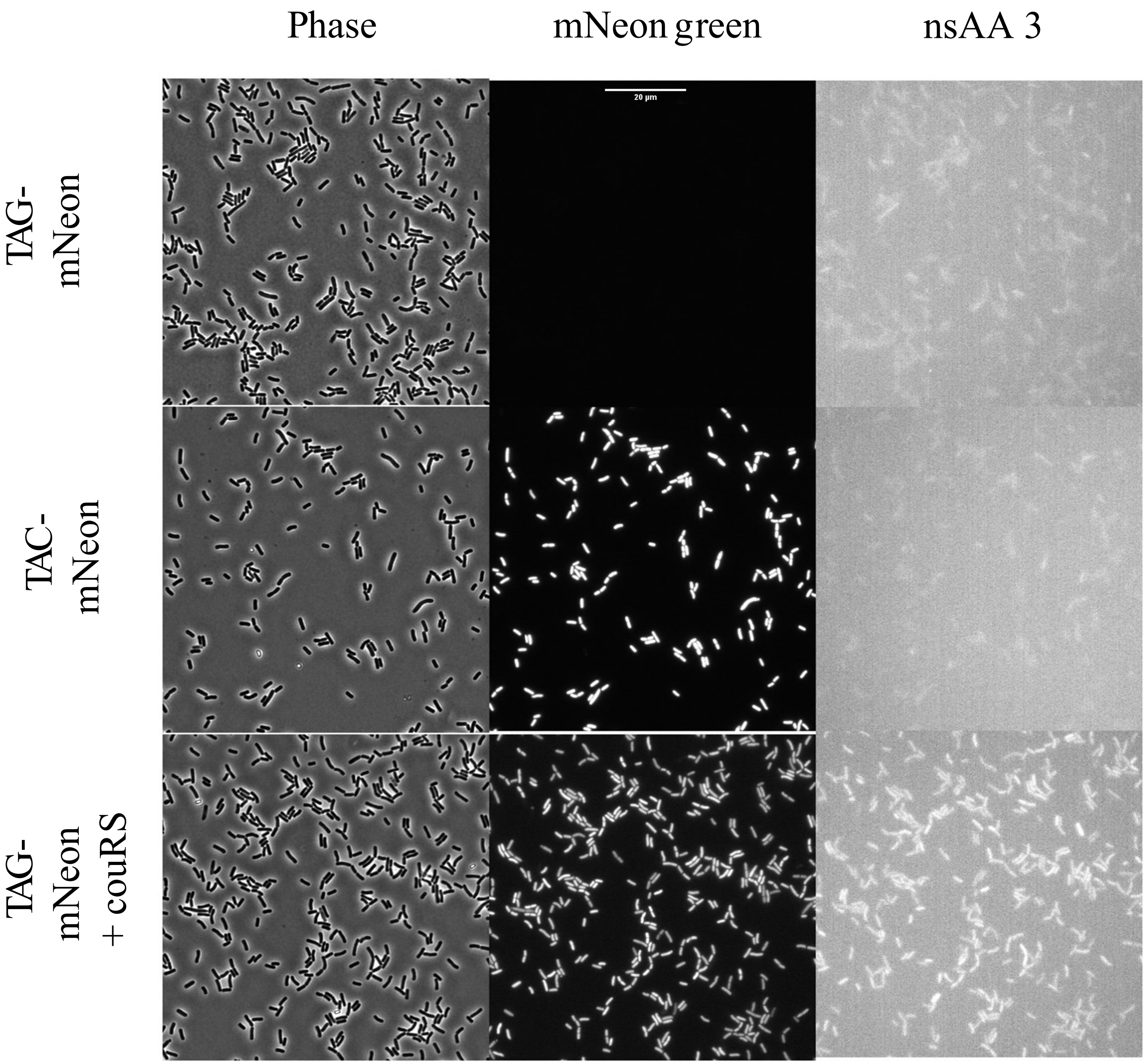

Supplemental Figure 3: Fluorescence imaging using CouAA Images of *B. subtilis* containing mNeongreenM10S reporters with and without a stop codon and with and without CouRS taken in phase, GFP (mNeongreen), and DAPI (nsAA 3) wavelengths. nsAA 3 was supplied to all conditions. CouRS was co-expressed with cognate tRNA. Each column has identical imaging conditions and brightness/contrast settings. A 20 uM scale bar is shown at the top of the figure, and applies to all panels.

Supplemental Figure 4

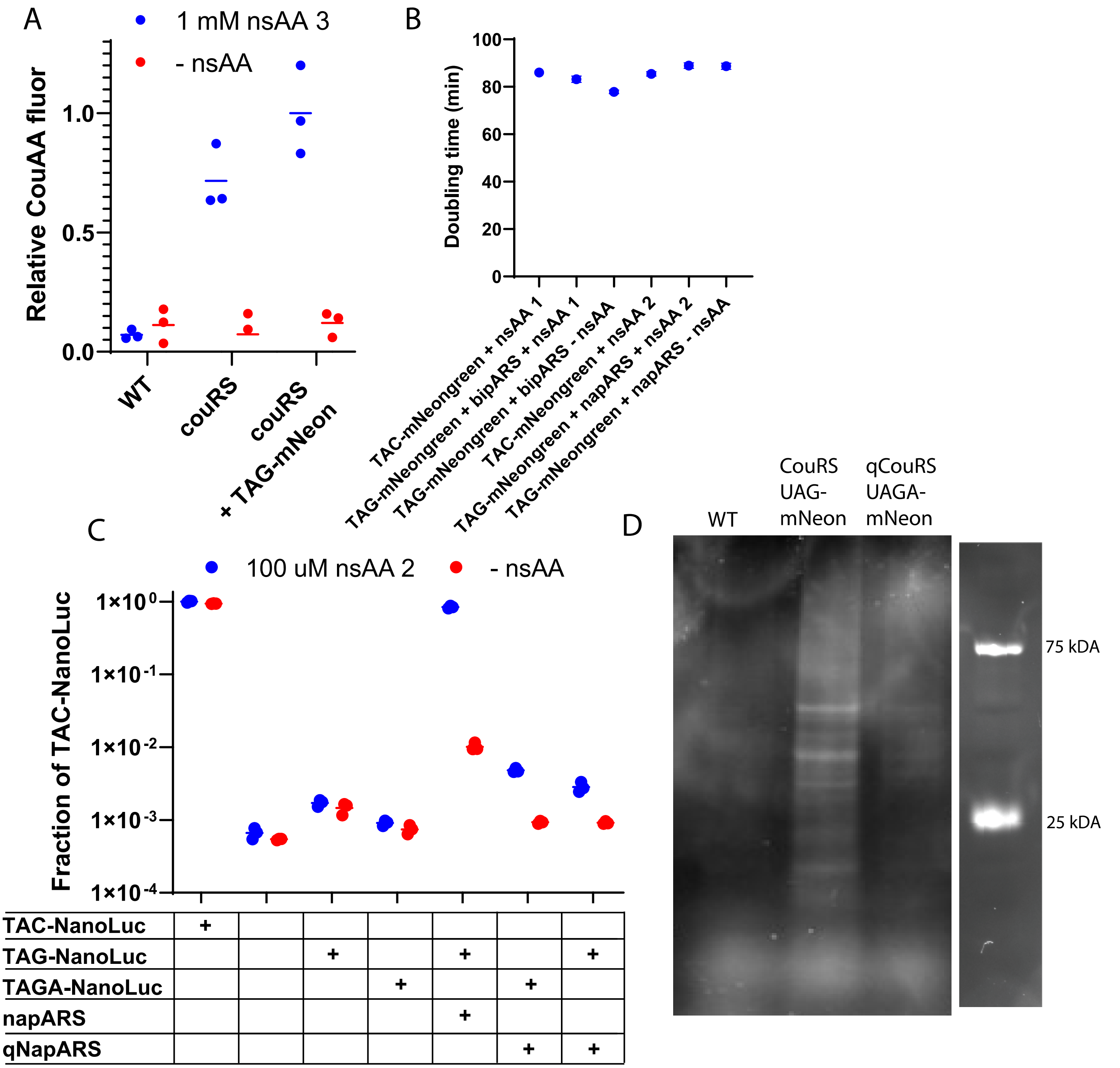

Supplemental Figure 4: Genomic incorporation and doubling times. A) Fluorescence of the CouAA amino acid remaining in bulk cells after washing. Three independent biological replicates shown with mean as a horizontal line, normalized to fraction of TAG signal. CouRS was co-expressed with cognate tRNA. B) Doubling times of *B. subtilis* cells with and without synthetase and 100 uM of nsAA. Single timecourses were fit to exponential growth curves, with 95% confidence intervals from the fits shown as error bars C) High-sensitivity detection of nanoluciferase reporter with UAC, UAG or UAGA codons inserted at position 2 of NanoLuc. Triplicates shown with mean as horizontal bar. Normalized to fraction of expression of UAC construct. D) Whole-cell lysate of cells grown with CouAA run on SDS-page gels and imaged for CouAA fluorescence in the proteome.

Supplemental Table 1

#	Gene Name	Percentage of nsAA-containing UAG proteins	Gene function	Essential?	Ends with TAGA?
1	alsS	13.1	acetolactate synthase	No	No
2	purB	10.0	purine biosynthesis	No	Yes
3	rplX	5.1	ribosomal protein L24	Yes	No
4	rpsG	4.5	ribosomal protein S7	Yes	No
5	yfmC	4.0	iron uptake	No	No
6	secA	3.6	protein secretion	No	No
7	accD	3.4	acetyl-CoA carboxylase	Yes	Yes
8	ytpR	3.2	unknown (possible tRNA synthetase)	No	Yes
9	atpG	3.0	ATP synthase	No	Yes
10	pyrAA	3.0	pyrimidine biosynthesis	No	Yes
11	ilvD	2.9	biosynthesis of branched-chain amino acids	No	Yes
12	yloV	1.7	fatty acid kinase	No	Yes
13	dapB	1.6	biosynthesis of lysine and peptidoglycan	Yes	Yes
14	ywlF	1.5	ribose-5-phosphate isomerase	No	No
15	ywhA	1.4	unknown (probable transcriptional regulator)	No	No
16	yaaK	1.4	unknown (possible DNA-binding protein)	No	No
17	murE	1.3	peptidoglycan precursor biosynthesis	Yes	No
18	ylxF	1.2	unknown	No	No
19	noc	1.2	control of cell division	No	Yes
20	ylxM	1.1	presecretory protein translocation	No	No
21	aroC	1.1	biosynthesis of aromatic amino acids	No	Yes
22	gsaB	1.0	biosynthesis of heme, modification of Efp	No	Yes
23	yeeB	1.0	unknown (possible helicase)	No	No

Supplemental Table 1: High-abundance proteins containing nsAAs. Proteins containing UAG stop codons ranked by abundance in the nsAA-incorporation enrichment by click-pulldown and quantified by mass-spectrometry. Only proteins above 1% abundance are shown and make up 71% of all nsAA-containing proteins found in the enrichment.

Supplemental Figure 5

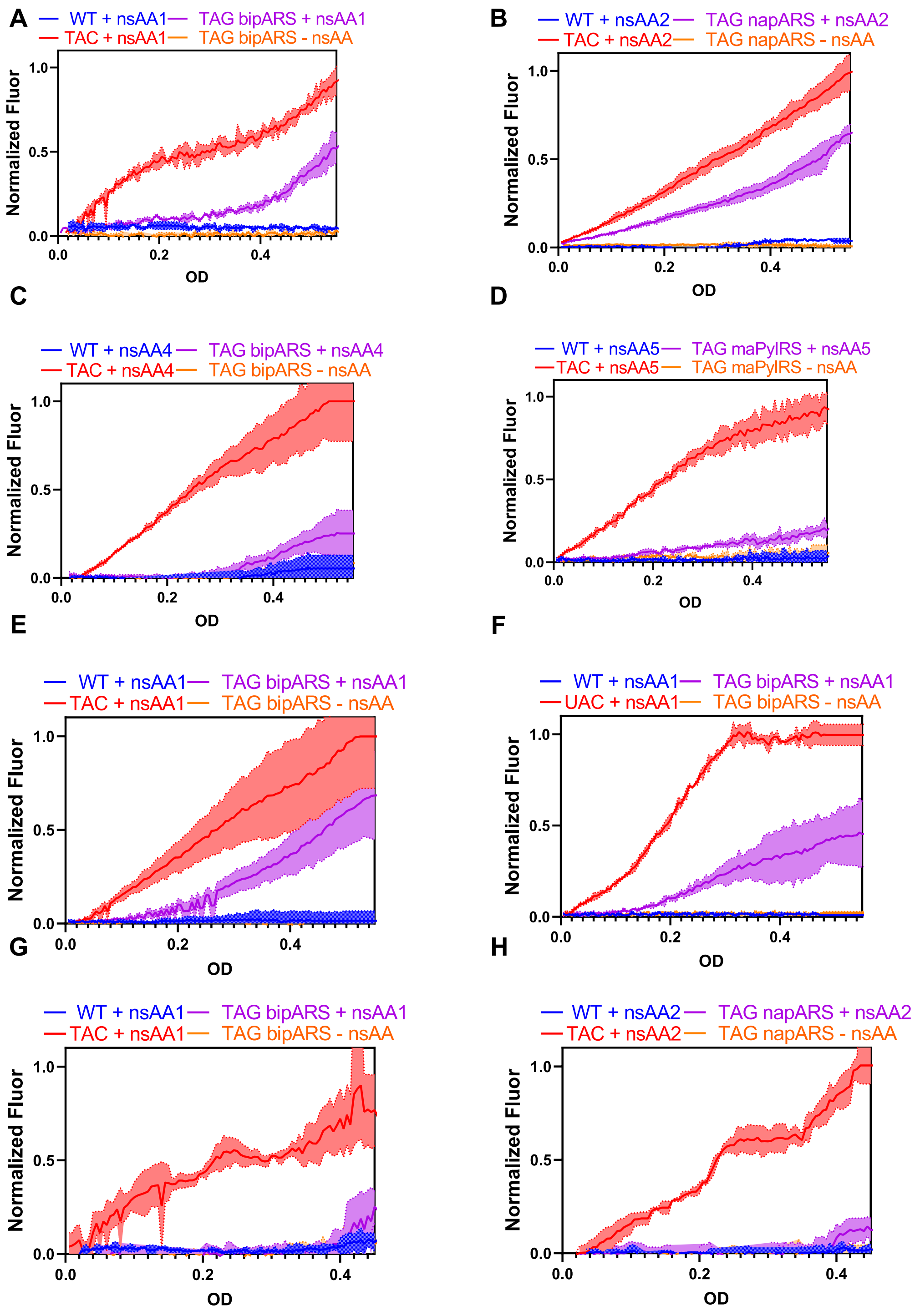

Supplemental Figure 5: Fluorescence vs. OD time courses for various nsAAs. Fluorescence and OD curves for different nsAAs and media conditions. A-D) S750 minimal media. E) S750 minimal media plus 1% w/w Pluronic F-68 F) S750 media modified to replace all amino acids with 0.3% buffered ammonium sulfate. G-H) CH rich media. Shaded area represents standard deviation between three biologically independent cultures at each time point.

Supplemental Figure 6

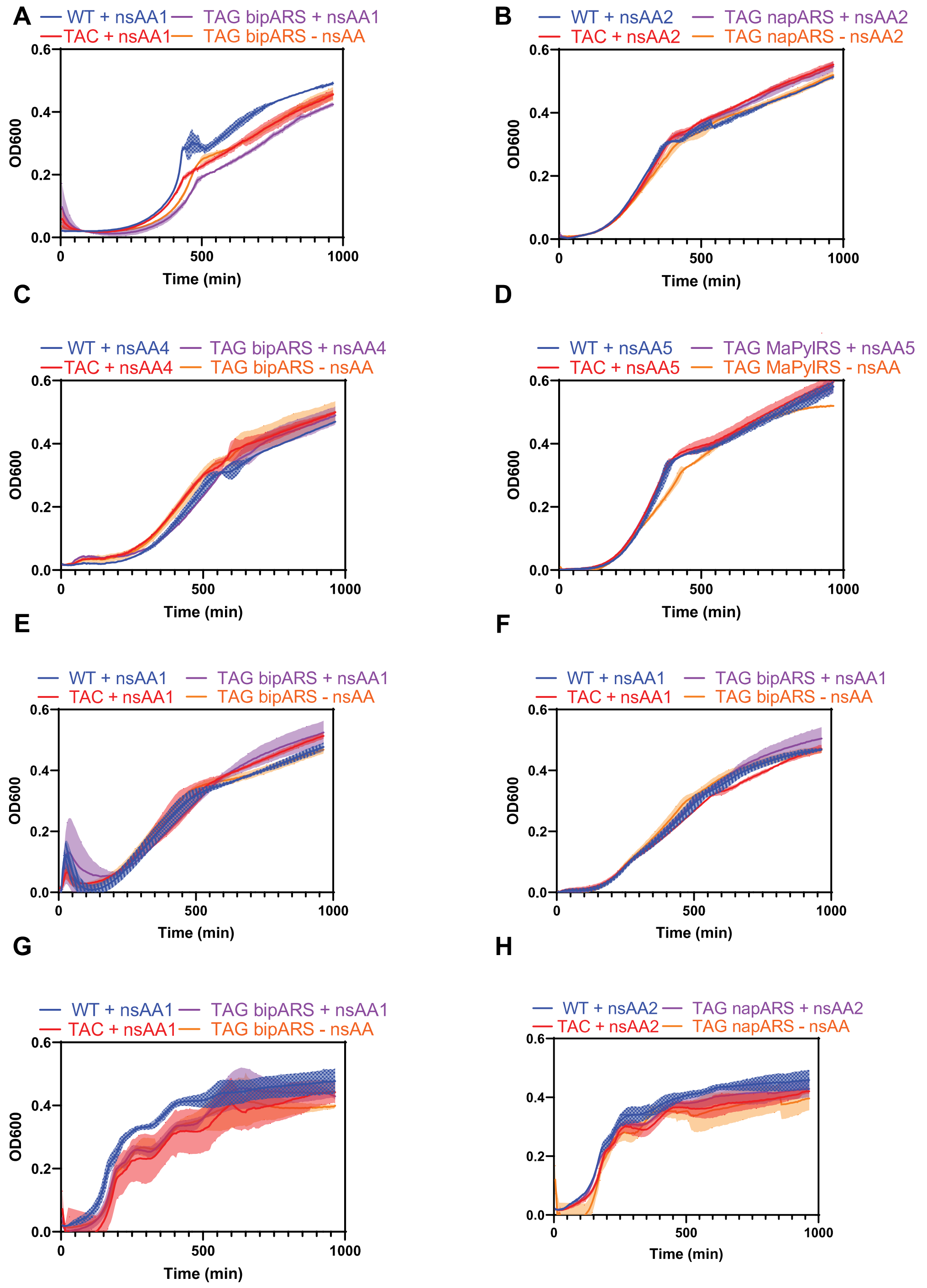

Supplemental Figure 6. OD time courses for various nsAAs. OD curves for different nsAAs and media conditions shown in Supplemental figure 5. A-D) S750 minimal media. E) S750 minimal media plus 1% w/w Pluronic F-68 F) S750 media modified to replace all amino acids with 0.3% ammonium sulfate. G-H) CH rich media. Shaded area represents standard deviation between three biologically independent cultures at each time point.

Supplemental Figure 7

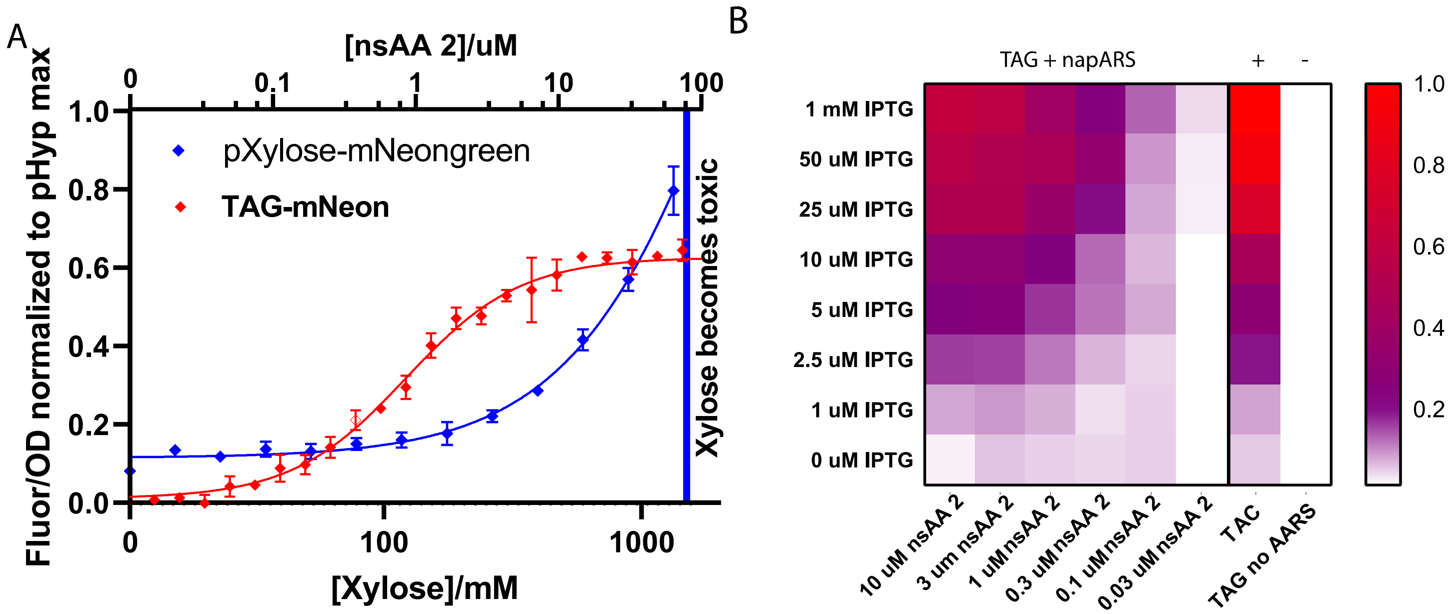

Supplemental Figure 7: Extended titration data. A) Titration and sigmoidal fit of pXylose-mNeongreen, with a nsAA 2-induced TAG-mNeongreen from Figure 4A overlaid. 3M xylose significantly reduces growth rate. N=3 biologically independent cultures, and error bars represent standard deviation. B) 2-dimensional titration data, alternate display for the dataset in figure 4B.

Supplemental Figure 8

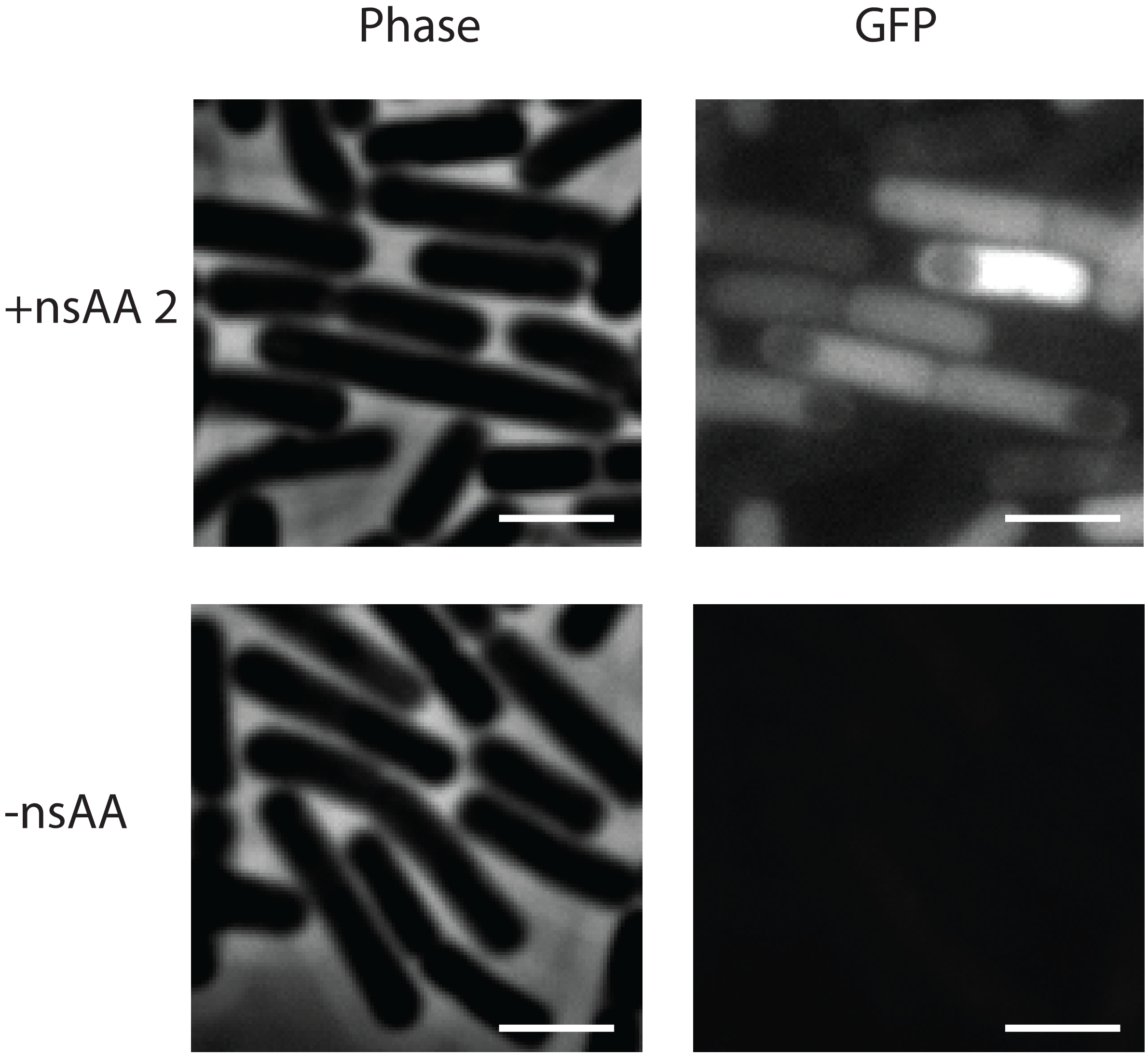

Supplemental Figure 8: Incorporation of nsAA 2 in sporulating *B. subtilis* cells. Cells with GFP(F27TAG) under control of a mother-cell specific promoter, P_{spoIIE}, were induced to sporulate by resuspension. At the time of resuspension, the culture was split in two, and nsAA 2 was added to the experimental sample. An example image of sporulating cells at 150 minutes after resuspension shows the GFP signal in the mother cell compartment of engulfed sporulating cells. GFP images shown were taken with identical acquisition times and settings. The control sample shows fluorescent signal at background levels when nsAA 2 is not present. Scale bar shown is 2 microns.

Supplemental Table 2

Alias	5' -> 3' Sequence
oDS066	TAGGCTGATGCTCCGCTC
oDS068	TTCAGGTCGGAAACAGGGAGCACTGGTCAAC
oDS074	CGAGGGAGCAGAATTGATTTTGCCGCTTAAC
oDS076	CGAGGGAGCAGAAGAGAGAATATAAAAAGCCAGATTATTAATC
oDS078	AGGAGGAACTCATATGGATGAATTTGAGATGATAAAACGTAACACG

oDS086	TCAGCGGCGTTTTCAAAAAGCTGGTCTGATATG
oDS088	TCAGCGGCGTTTTAACCTTTTAGGAGCCAGC
oDS091	ACGCAGTCTATATCCGGCGGTAGTTCAGCAG
oDS092	AATAAGTTAAGATCCGGCGGTAGTTCAGCAG
oDS093	AAAGCTTGCGCCTGCATGCAAAAAAGCCTGCTC
oDS094	CTTTTTTGCATGCAGGCGCAAGCTTTAATCC
oDS096	GGGCAAGGCTAGACGGGA
oDS098	AACGAGTATGTGATTCTGCTCCCTCGCTCAG
oDS099	AGGAAATCCATTACAGGGAGCACTGGTCAAC
oDS101	CCAGTGCTCCCTGTAATGGATTTCCTTACGC
oDS102	CAAATATCTGCATGGTAGTTCCTCCTTAAAG
oDS106	CTTTCGAAACCATAAGCTCTTGAACGAACAAC
oDS107	CGTTCAAGAGCTTATGGTTTCGAAAGGAGAG
oDS108	GAATCCATCCAAATCACTTATAGAGTTCATCCATAC
oDS109	ACTCTATAAGTGATTTGGATGGATTCAGCCC
oDS110	TCTTGACACTCCTTATTTGATTTTTTG
oDS135	TCTTGACACTCCTTATTTGATTTTTTGAAGACTTACTTCGG
oDS213	ATCTCAAATTCATCCATATGAGTTCCTCCTTACGCACTACATTTA
oDS215	AACGCGGCGTTTTTTAAAGTCTCTTCCGTATTGGCTCCA
oDS296	AGAGGAGGATAATTCTGCTAGCCTCCCAGCG
oDS297	GGGAGGCTAGCAGAATTATCCTCCTCTCCTTTCG
oDS402	CATGGTAGTTCCTCCTTAAAGCTTAATTGTTATCC
oDS406	TTTGGATGGATTCAGCCCGATTG
oDS438	CAAGGCTAGACGGGACTTACCGAAAGAAACCATCAA
oDS448	AGGCGCAAGCTTTAATCCCGGCAAC
oDS449	GTTGACCAGTGCTCCCTGATTCTCCTCCTTGTTCTCTTAGCCC
oDS450	CAAGGAGGAGAATCAGGGAGCACTGGTCAACTACCG
oDS451	GCATCAGCCTAGCATGCAAAAAAGCCTGCTCGTTGAG
oDS452	GAGCAGGCTTTTTTGCATGCTAGGCTGATGCTCCGCTCGATAT
oDS453	TTTCCGACCTGAACCATGCCGATG
oDS454	GTCTTCAAAAAATCAAATAAGGAGTGTCAAGATTCTGCTCCCTCGCTCAGTACCG
oDS455	TCACATACTCGTTTCCAAACGGATCATACAA
oDS457	TCTTTCGGTAAGTCCCGTCTAGCCTTGCCCTCACTTATAGAGTTCATCCATACCCATC
oDS608	GGTGTTCCGGGTGTAGGTAAGGGTGGAGGGACGGTTTCGAAAGGAGAGGAGGATAATTCT
oDS612	CCTTGTAATCATGATGATGATGATGGTGCTTATAGAGTTCATCCATACCCATCACGTCTG
oDS613	AAGGACCACGACATCGACTACAAAGACGATGATGATAAATGAGGGCAAGGCTAGACGGGA
oDS614	CTTGTAGTCGCCGTCGTGATCCTTGTAATCATGATGATGATGATGGTGCTTATAG
oDS615	ATTACAAGGATCACGACGGCGACTACAAGGACCACGACATCGACTACAAAGACGA
oDS618	AGCTTGACGGAAGTCAATATTGGTAAAGGAGGTTTTTTTATGAGCAAAGGTCCTGGCAAG
oDS619	CTTTACCAATATTGACTTCCGTCAAGCTTAATTGTTATCCGCTCACAATTACACACATTA
oDS624	CACAATTCCACACATTATGCCACACCTTGTAGATAAAGTC
oDS628	CCCAGCAATCGGGCTGAATCCATCCAAATTACGCCAGAATCCGCTCGCAAA
oDS636	GTTCCTCCTTAAGCTTAATTGTTATCCGCTCACAATT
oTS170	AGCGATGGCGAAGCAATACGGCTAGGAAAGCCAAGAAGTATTAAATC
oTS172	GAATGATCTCTGATTTGAAAAGCATGTAGGAAGGTGCTTCAA
oWM28	ATGGTTTCGAAAGGAGAGGAGG
oAB139	tatcctcctctcctttcgaaaccatGCTAAATCCTCCTAATCTGCCGAATG
oAB197	AGGCGCAAGCTTTAATCCCG

oSW251	gtagttgaccagtgctccctgATTCTCCTCCTTGTTCTCTT
oSW40	CAGGGAGCACTGGTC
oSW42	TTCTGCTCCCTCGC
oAB76	gaacggtactgagcgagggagcagaaGTATTTGTTTCCGGTTTC
oWM248	gcccatatcgagcggagcatcagcTTAGCCGCGTTTATTACGGT
oSW254	TAGGCTGATGCTCCGCT
oAB196	CCGACCTGAACCATGCCGAT
oSW250	AGGCGCAAGCTTTAATC
oAB13	ccagtaccgatttctgccatGCTAAATCCTCCTAATCTGCCGAATG
oWM24	ATGGCAGAAATCGGTACTG
oWM248	gcccatatcgagcggagcatcagcTTAGCCGCGTTTATTACGGT
oVP005	ggtgatgttaatgggcacaaatAGtctgtcagtggagagggt

Supplemental Table 2: Primers used in this study. Details on how primers are used is contained in attached strain construction excel document.

Methods

References:

1. Scheidler, C. M., Vrabel, M. & Schneider, S. Genetic Code Expansion, Protein Expression, and Protein Functionalization in Bacillus subtilis. *ACS Synth. Biol.* **9**, 486–493 (2020).
2. Salis, H. M., Mirsky, E. A. & Voigt, C. A. Automated design of synthetic ribosome binding sites to control protein expression. *Nature Biotechnology* **27**, 946–950 (2009).
3. Bisson-Filho, A. W. *et al.* Treadmilling by FtsZ filaments drives peptidoglycan synthesis and bacterial cell division. *Science* **355**, 739–743 (2017).
4. Subramanian, S., Gao, X., Dann, C. E. & Kearns, D. B. MotI (DgrA) acts as a molecular clutch on the flagellar stator protein MotA in Bacillus subtilis. *PNAS* **114**, 13537–13542 (2017).
5. England, C. G., Ehlerding, E. B. & Cai, W. NanoLuc: A Small Luciferase is Brightening up the Field of Bioluminescence. *Bioconjug Chem* **27**, 1175–1187 (2016).
6. Kunjapur, A. M. *et al.* Engineering posttranslational proofreading to discriminate nonstandard amino acids. *PNAS* **115**, 619–624 (2018).
7. Youngman, P. J., Perkins, J. B. & Losick, R. Genetic transposition and insertional mutagenesis in Bacillus subtilis with Streptococcus faecalis transposon Tn917. *Proc Natl Acad Sci U S A* **80**, 2305–2309 (1983).
8. Gibson, D. G. *et al.* Enzymatic assembly of DNA molecules up to several hundred kilobases. *Nature Methods* **6**, 343–345 (2009).
9. Zhang, M. S. *et al.* Biosynthesis and genetic encoding of phosphothreonine through parallel selection and deep sequencing. *Nature Methods* **14**, 729–736 (2017).
10. Squyres, G. R. *et al.* Dynamics of bacterial cell division: Z ring condensation is essential for cytokinesis. *bioRxiv* 2020.06.30.180737 (2020) doi:10.1101/2020.06.30.180737.
11. Harwood, C. R. & Cutting, S. M. *Molecular biological methods for Bacillus*. (Wiley, 1990).
12. Burton, B. M., Marquis, K. A., Sullivan, N. L., Rapoport, T. A. & Rudner, D. Z. The ATPase SpoIIIE Transports DNA across Fused Septal Membranes during Sporulation in Bacillus subtilis. *Cell* **131**, 1301–1312 (2007).
13. Sysoeva, T. A., Zepeda-Rivera, M. A., Huppert, L. A. & Burton, B. M. Dimer recognition and secretion by the ESX secretion system in Bacillus subtilis. *PNAS* **111**, 7653–7658 (2014).

14. Huppert, L. A. *et al.* The ESX System in Bacillus subtilis Mediates Protein Secretion. *PLOS ONE* **9**, e96267 (2014).
