# Supplementary material for: Designing efficient genetic code expansion in Bacillus subtilis to gain biological insights
Source: Nat Commun. 2021 Sep 14;12:5429. doi: 10.1038/s41467-021-25691-4 (PMC8440579; doi:10.1038/s41467-021-25691-4)
Supplement: Supplementary file 2 — Reporting Summary [file 41467_2021_25691_MOESM2_ESM.pdf]

# nature portfolio

Corresponding author(s): Aditya Kunjapur, Ethan Garner, George Church

Last updated by author(s): Jun 29, 2021

# Reporting Summary

Nature Portfolio wishes to improve the reproducibility of the work that we publish. This form provides structure for consistency and transparency in reporting. For further information on Nature Portfolio policies, see our Editorial Policies and the Editorial Policy Checklist.

## Statistics

For all statistical analyses, confirm that the following items are present in the figure legend, table legend, main text, or Methods section.

| n/a | Confirmed | |
|---|---|---|
| ☐ | ☒ | The exact sample size ($n$) for each experimental group/condition, given as a discrete number and unit of measurement |
| ☐ | ☒ | A statement on whether measurements were taken from distinct samples or whether the same sample was measured repeatedly |
| ☒ | ☐ | The statistical test(s) used AND whether they are one- or two-sided<br>*Only common tests should be described solely by name; describe more complex techniques in the Methods section.* |
| ☒ | ☐ | A description of all covariates tested |
| ☒ | ☐ | A description of any assumptions or corrections, such as tests of normality and adjustment for multiple comparisons |
| ☐ | ☒ | A full description of the statistical parameters including central tendency (e.g. means) or other basic estimates (e.g. regression coefficient) AND variation (e.g. standard deviation) or associated estimates of uncertainty (e.g. confidence intervals) |
| ☒ | ☐ | For null hypothesis testing, the test statistic (e.g. $F$, $t$, $r$) with confidence intervals, effect sizes, degrees of freedom and $P$ value noted<br>*Give P values as exact values whenever suitable.* |
| ☒ | ☐ | For Bayesian analysis, information on the choice of priors and Markov chain Monte Carlo settings |
| ☒ | ☐ | For hierarchical and complex designs, identification of the appropriate level for tests and full reporting of outcomes |
| ☒ | ☐ | Estimates of effect sizes (e.g. Cohen's $d$, Pearson's $r$), indicating how they were calculated |

*Our web collection on statistics for biologists contains articles on many of the points above.*

## Software and code

Policy information about availability of computer code

| | |
|---|---|
| Data collection | Gen5 (basic version, BioTek) was used to collect all plate reader data. XCalibur Software 4.3 (Thermo Fisher Scientific) was used to collect all mass-spectrometry data. NIS-Elements software version 5.02.01 was used to generate all microscopy elements except the sporulation images, which were generated with Zen 2.0 software (Zeiss). Chemidoc analysis was done with Image Lab Touch 2.2. |
| Data analysis | Sequest (Proteome Discoverer, Thermo Fisher Scientific) was used to analyze mass-spectrometry data. Microsoft Excel (365) was use d to analyze plate reader data, and Graphpad Prism 9 was used to generate plots, calculate standard deviations and perform best-fit titration analysis. Division microscopy data was analyzed with Matlab, version 2018b with Morphometrics and DeepCell packages, as well as FIJI, version 1.53, including the plugin TrackMate, and custom code available at https:// bitbucket.org/garnerlab/squyres-2020/src/ master/. Gel and microscopy data was analyzed and prepared for presentation with ImageJ with FIJI, version 1.53. |

For manuscripts utilizing custom algorithms or software that are central to the research but not yet described in published literature, software must be made available to editors and reviewers. We strongly encourage code deposition in a community repository (e.g. GitHub). See the Nature Portfolio guidelines for submitting code & software for further information.

## Data

Policy information about availability of data

All manuscripts must include a data availability statement. This statement should provide the following information, where applicable:

- Accession codes, unique identifiers, or web links for publicly available datasets
- A description of any restrictions on data availability
- For clinical datasets or third party data, please ensure that the statement adheres to our policy

All the DNA sequence data used in this manuscript is provided in the Supplementary Information files, and all raw numerical data and gel images obtained from measurements in this study are also provided as Supplementary files associated with this manuscript. Source data are provided with this paper

this paper.

# Field-specific reporting

Please select the one below that is the best fit for your research. If you are not sure, read the appropriate sections before making your selection.

[x] Life sciences  [ ] Behavioural & social sciences  [ ] Ecological, evolutionary & environmental sciences

For a reference copy of the document with all sections, see nature.com/documents/nr-reporting-summary-flat.pdf

# Life sciences study design

All studies must disclose on these points even when the disclosure is negative.

| | |
|---|---|
| Sample size | We applied the precedent in the field of N=3 for plate reader, time-course and nsAA concentration liquid chromatography/ mass-spectrometry experiments, which generally contained low variation among sample replicates within an experiment. No calculation was used to determine sample size. However, these experiments were repeated and exhibited very similar results. The other experiments, including mass spectrometry experiments, microscopy experiments and crosslinking used a sample size of N=1. We did not use a calculation to determine sample size, and we did not always repeat experiments. However, the analytical techniques, analysis methods, and/or controls that we used give us high confidence in the results that were obtained. |
| Data exclusions | Many more experiments were performed than are presented. Some of these were replicate experiments with similar results, and many were negative experiments with attempts to use different conditions or nonstandard amino acids tor various purposes. For instance, click-labelling of incorporated pAzF on the cell surfaces does not get around background incorporation, the standard MaPylRS cannot efficiently incorporate the photocrosslinker AbK, external Apidaecin peptides do not improve nsAA incorporation in B.subtilis, knocking out PrmC improves nsAA incorporation efficiency but is too toxic to use for most applications, and more.These experiments are excluded tor the sake of concision and because generating publication-quality data tor these negative results would be unacceptably costly in time and materials. |
| Replication | Typically plate reader experiments were conducted multiple times, often with slightly different experimental or procedural elements in an attempt to optimize data collection. In general, the findings observed in this paper were observed in all experiments, with exceptions where cells didn't grow or contamination was believed to have occurred.Mass-spectrometry, gel blot and microscopy data was sometimes replicated in this fashion, as noted in the Statistics and Reproducibility section of the manuscript. |
| Randomization | Experimental samples organized into different experimental groups were generated from the same source.If one strain was exposed to +nsAA and -nsAA conditions, a single culture of that strain was split immediately before nsAA was added. |
| Blinding | All experiments were unblinded, and strains referred to by their strain numbers throughout experimental procedures, data collection and analysis.This is due to the difficulty of fully blinding rapidly-iterated experiments and the unlikeliness that researcher bias in treatment of bacterial strains would result in the replicable effect sizes observed in this manuscript. |

# Reporting for specific materials, systems and methods

We require information from authors about some types of materials, experimental systems and methods used in many studies. Here, indicate whether each material, system or method listed is relevant to your study. If you are not sure if a list item applies to your research, read the appropriate section before selecting a response.

## Materials & experimental systems

| n/a | Involved in the study |
|---|---|
| [ ] | [x] Antibodies |
| [x] | [ ] Eukaryotic cell lines |
| [x] | [ ] Palaeontology and archaeology |
| [x] | [ ] Animals and other organisms |
| [x] | [ ] Human research participants |
| [x] | [ ] Clinical data |
| [x] | [ ] Dual use research of concern |

## Methods

| n/a | Involved in the study |
|---|---|
| [x] | [ ] ChIP-seq |
| [x] | [ ] Flow cytometry |
| [x] | [ ] MRI-based neuroimaging |

## Antibodies

| | |
|---|---|
| Antibodies used | custom rabbit polyclonal anti-yukE antibiodies and anti-sigA antibodies. goat anti-rabbit antibodies conjugated with horseradish peroxidase (HRP) wre used for secondary. |
| Validation | Antibodies were used and verified in Sysoeva, T.A, Zepeda-Rivera, M.A., Huppert, L.A.& Burton, B.M .Dimer recognition and secretion by the ESX secretion system in Bacillus subtilis.PNAS 111, 7053-7058 (2014) & Huppert, L.A.et al.The ESX System in Bacillus subtilis Mediates Protein Secretion.PLOS ONE 9, e96267 (2014).<br>Goat anti-rabbit come from Abcam, catalog # ab6721 |
