# Supplementary material for: Designing efficient genetic code expansion in Bacillus subtilis to gain biological insights
Source: Nat Commun. 2021 Sep 14;12:5429. doi: 10.1038/s41467-021-25691-4 (PMC8440579; doi:10.1038/s41467-021-25691-4)
Supplement: Supplementary file 4 — Description of Additional Supplementary Files [file 41467_2021_25691_MOESM4_ESM.pdf]

Title: Supplemental Movie 1

Description: Titration of MciZ expression and its effects on FtsZ filaments in vivo. Cells expressing mNeonGreen-FtsZ were imaged at 1-second intervals for 100 seconds by TIRF microscopy. The concentration of nsAA 2 added is indicated in each panel. In each case, the UAG-MciZ construct was induced with 100 µM IPTG. The movie is displayed at 30 frames per second (30x actual speed). Scale bar: 2 µm.
